# Chemoenzymatically prepared konjac ceramide inhibits NGF-induced neurite outgrowth by a semaphorin 3A-like action

**DOI:** 10.1016/j.bbrep.2015.11.016

**Published:** 2015-11-19

**Authors:** Seigo Usuki, Noriko Tamura, Shota Sakai, Tomohiro Tamura, Katsuyuki Mukai, Yasuyuki Igarashi

**Affiliations:** a Laboratory of Biomembrane and Biofunctional Chemistry, Graduate School of Advanced Life Science, Frontier Research Center for Post-Genome Science and Technology, Hokkaido University, Kita 21, Nishi 11, Kita Ward, Sapporo, Hokkaido 011-0021, Japan; b National Institute of Advanced Industrial Science and Technology (AIST), Sapporo, Hokkaido, Japan; c Healthcare Division, Daicel Corporation, Japan

**Keywords:** NGF, nerve growth factor, Cer, ceramide, GlcCer, glucosylceramide, kCer, konjac ceramide, C2Cer, N-acetyl-D-erythro-sphingosine, C16Cer, N-hexadecanoyl-D-erythro-sphingosine, C18Cer, N-octadecanoyl-D-erythro-sphingosine, C24Cer, N-tetracosanoyl-D-erythro-sphingosine, EGCase I, endoglycoceramidase I, CRMP2, collapsin response mediator protein 2, pCRMP2, phospho-collapsin response mediator protein 2, LDH, lactate dehydrogenase, TBEA, trypan blue exclusion assay, CCK-8, cell counting kit 8, Sema 3A, semaphorin 3A, CBB, Coomassie Briliant Blue, BSA, bovine serum albumin, PBS, phosphate-buffered saline, DMEM, Dulbecco’s modified Eagle's medium, Ceramide, Konjac, NGF, Semaphorin 3A, Neurite outgrowth, CRMP2

## Abstract

Dietary sphingolipids such as glucosylceramide (GlcCer) are potential nutritional factors associated with prevention of metabolic syndrome. Our current understanding is that dietary GlcCer is degraded to ceramide and further metabolized to sphingoid bases in the intestine. However, ceramide is only found in trace amounts in food plants and thus is frequently taken as GlcCer in a health supplement. In the present study, we successfully prepared konjac ceramide (kCer) using endoglycoceramidase I (EGCase I). Konjac, a plant tuber, is an enriched source of GlcCer (kGlcCer), and has been commercialized as a dietary supplement to improve dry skin and itching that are caused by a deficiency of epidermal ceramide. Nerve growth factor (NGF) produced by skin cells is one of the itch factors in the stratum corneum of the skin. Semaphorin 3A (Sema 3A) has been known to inhibit NGF-induced neurite outgrowth of epidermal nerve fibers. It is well known that the itch sensation is regulated by the balance between NGF and Sema 3A. In the present study, while kGlcCer did not show an *in vitro* inhibitory effect on NGF-induced neurite outgrowth of PC12 cells, kCer was demonstrated to inhibit a remarkable neurite outgrowth. In addition, the effect of kCer was similar to that of Sema 3A in cell morphological changes and neurite retractions, but different from C2-Ceramide. kCer showed a Sema 3A-like action, causing CRMP2 phosphorylation, which results in a collapse of neurite growth cones. Thus, it is expected that kCer is an advanced konjac ceramide material that may have neurite outgrowth-specific action to relieve uncontrolled and serious itching, in particular, from atopic eczema.

## Highlights

• We prepared konjac ceramide (kCer) from konjac GlcCer using endoglycoceramidase I.
• kCer inhibits NGF-induced neurite outgrowth by semaphorin 3A-like action.
• Konjac GlcCer does not show NGF-induced neurite outgrowth such as kCer.

## 1 Introduction

Epidermal neurotrophins play an important role for proliferation of structural skin cells such as keratinocytes, fibroblasts, and mast cells, and regulate peripheral innervation [1]. Cutaneous functions of nerve growth factor (NGF) have been well studied and known to maintain cutaneous innervation and cell survival by protecting sensory neurons via retrograde neurotrophic signaling [2]. It is also well known that the cutaneous level of NGF is regulated as an autocrine and paracrine growth factor in all skin cells [3], [4]. An excessive amount of NGF shows an additional function as a pain or itch sensitizer via the cutaneous innervation system. Epidermal NGF elevation stimulates skin cells, inducing overproduction of NGF, sensitizing the nociceptive nerve endings of sensory neurons, enhancing neurite outgrowth, and resulting in structural changes of the innervation system. Such extended neurites penetrate into the superficial layer of the skin and indicate hypersensitivity for external stimuli [5]. Thus, the undesired NGF causes extension of itch-causing nerve fibers and severe or long-lasting, itching skin, such as atopic eczema. To prevent external stimuli from inducing hypersensitivity of sensory neurons, epidermal ceramide is crucial for maintenance of homeostasis of an epidermal barrier. Epidermal ceramides are believed to be derived from pools of sphingomyelin and glucosylceramide (GlcCer) from differentiated keratinocytes via glucosylceramide synthase [6] and sphingomyelin synthase [7], respectively, and function as an epidermal barrier of water permeability and external stimuli [8].

When this ceramide-enriched superficial structure is injured or acquires developmental dysfunctions, the external stimuli can penetrate into the barrier and irritate the keratinocytes, or mast cells and fibroblasts, enhancing production and secretion of NGF and inflammatory cytokines [1].

**Fig. 1:**
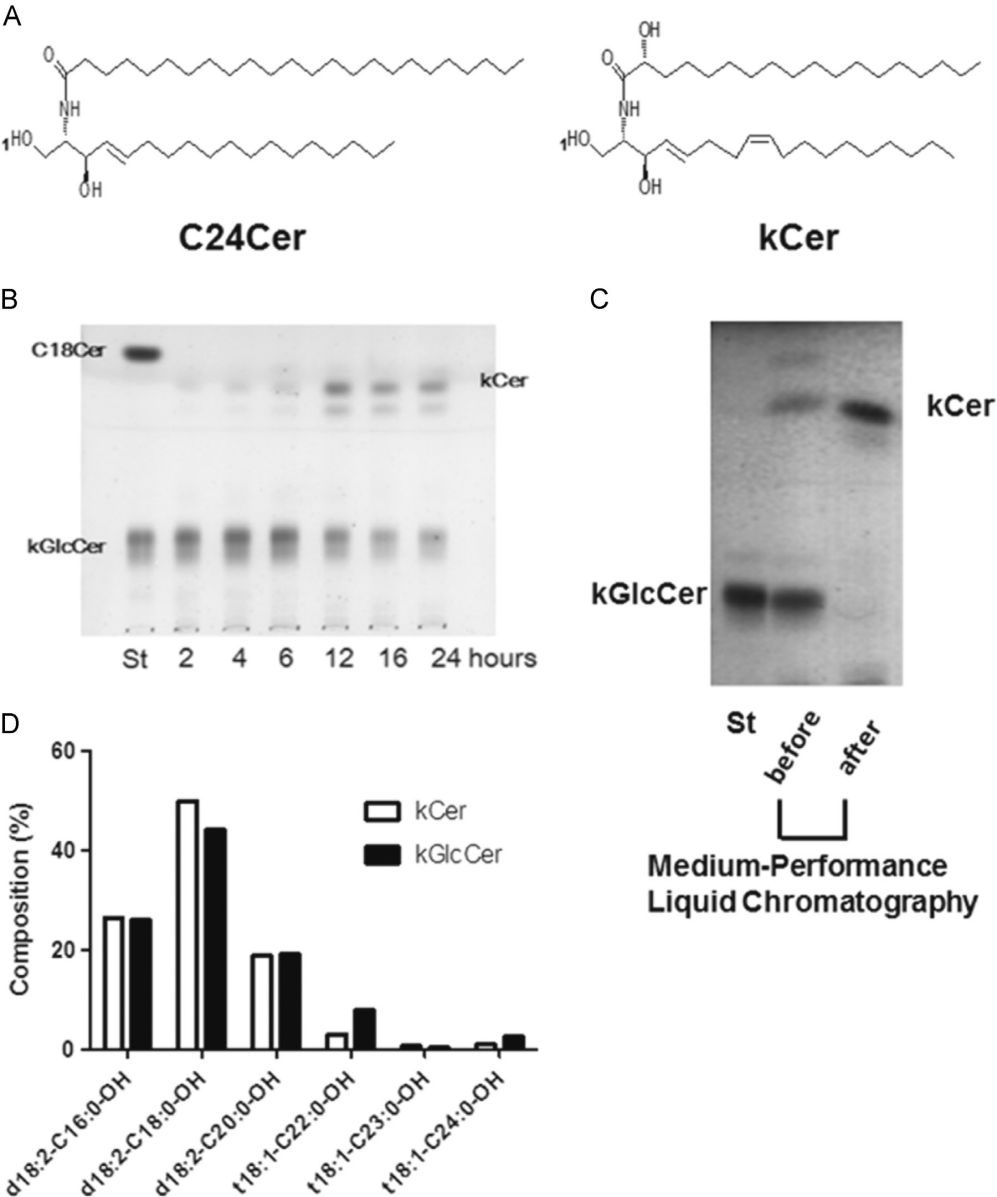
(A). Chemical structure of typical ceramide species: C24Cer, one of the major species in human epidermis, and kCer, one of the major species in *Amorphophallus konjac*. (B) Time course of EGCase I reaction. The enzyme reaction was performed at 37 °C in sodium acetate buffer pH 5.0 with kGlcCer, 135 nmol; EGCase I, 70.3 mU. After the enzyme reaction, Bligh–Dyer extracts were prepared and were examined by development of TLC using chloroform:methanol:acetic acid (65:10:0.3, v/v) as solvent. TLC plates were sprayed with 10% cupric sulfate in 8% phosphoric acid, and heated at 180 °C. Plates were generally recorded using a photo scanner. The area density was quantitated using JustTLC software. (C) Bligh–Dyer extracts were subjected to medium pressure liquid chromatography on a Hi-Flash S column and programmed with a linear gradient elution from chloroform:methanol:acetic acid (99:2:0.4, v/v) to chloroform:methanol:acetic acid (99:4:0.4, v/v) for 10 min. Column fractions were analyzed by TLC as shown. (D) Molecular species of kCer and kGlcCer after EGCase I reaction were analyzed by LC–ESI–MS/MS. No differences in the relative amounts of kCer and kGlcCer were seen in the combinations of sphingoid bases (d18:2, t18:1) and hydroxyl fatty acids (C16:0-OH, C18:0-OH, C20:0-OH, C22:0-OH, C23:0-OH, C24:0-OH).

Konjac (*Amorphophallus konjac K. Koch*) is a food plant that is abundant in GlcCer. The efficacy of konjac GlcCer (kGlcCer) in trans-epidermal water loss in mice and humans has been studied by Uchiyama et al. [9]. However, it remains to be elucidated whether kGlCer and its metabolites have a direct effect on itch-causing neurite outgrowth. In the present study, we examined whether kGlcCer and konjac ceramide (kCer, see Fig. 1A for its structure), which is a trace component of konjac extract, possess an inhibitory effect on NGF-induced neurite outgrowth. We chemoenzymatically prepared enough kCer using endoglycoceramidase I (EGCase I) [10] to perform cellular experiments. We found remarkable neurite retractions caused by kCer, but not by kGlcCer, in PC12 cells.

It is well known that extensions and retractions of skin sensory nerve fibers are regulated by the balance of nerve elongation factors such as NGF and nerve repulsion factors such as semaphorin 3A (Sema 3A) [11]. Our findings presented here indicate that kCer has a similar activity as Sema 3A, which is a growth cone-modulator protein, via CRMP2 phosphorylation, and competes with NGF in epidermal homeostasis [12].

## 2 Materials and methods

### 2.1 kCer preparation

GlcCer from konjac was commercially obtained from Nagara Science Co, Ltd. (Gifu, Japan). Thin-layer chromatography (TLC) analysis showed homogeneous purity of kGlcCer to be more than 99%. Ten aliquots of kGlcCer (135 nmol each) were incubated overnight (16–2 h) at 37 °C with 0.1 M sodium acetate buffer (pH 5.0) using recombinant EGCase I isolated from a mutant strain of *Rhodococcus erythropolis* L-88 containing pTip-EGCI plasmid as host cell [10], [13]. After EGCase I treatment, samples were processed using the Bligh–Dyer extraction method [14] and analyzed by TLC using chloroform:methanol:acetic acid, 65:10:0.3, (v/v) as solvent. Following chromatography, TLC plates were sprayed with 10% cupric sulfate in 8% phosphoric acid, heated at 180 °C and recorded with a photo scanner. The area density was quantitated using JustTLC software (SWEDAY, Sodra Sanby, Sweden). The post-reaction samples shown to contain kCer and kGlcCer by TLC were then extracted by the Bligh–Dyer method. Ten of the Bligh–Dyer extracts were pooled and dried by nitrogen gas. The pooled crude samples were subjected to medium pressure liquid chromatography on a Hi-Flash S column (Yamazen Corp., Osaka, Japan), and separated into two column fractions containing kCer and kGlcCer, eluted such that one column fraction contained kCer and one fraction contained kGlcCer. The column fraction containing kCer was detected by TLC analysis with cupric sulfate/phosphate reagent, pooled, and dried by nitrogen gas. The combined kCer was quantitated by TLC-densitoscanning as described above. The kCer thus prepared was used for *in situ* treatment on PC12 cells in culture.

### 2.2 Neurite outgrowth assay

Rat adrenal pheochromocytoma PC12 cells were obtained from JCRB Cell Bank (Osaka, Japan). Cells were cultured in Dulbecco's modified Eagle's medium (DMEM) supplemented with 10% fetal bovine serum (FBS), 5% horse serum (HS), and 10 ml/L penicillin–streptomycin solution (Wako Co, Osaka, Japan), maintained at 37 °C in a humidified chamber under an atmosphere of 95% air and 5% CO_2_. NGF (mNGF 2.5 S, Almone Labs, Jerusalem, Israel) was dissolved in 0.025% bovine serum albumin (BSA)/DMEM containing kCer (or other ceramides: C2Cer, C16Cer, C18Cer). For NGF-induced differentiation, PC12 cells were plated in poly-l-lysine-coated 24-well plates at 3.0×10^4^ cells /well and incubated in DMEM with 10% FBS/5% HS overnight. Cells were washed with fresh serum-free DMEM and incubated for 48 hours with 0.025% BSA/DMEM containing the ceramides in the presence or absence of NGF (100 ng/mL). After removing the supernatant by suction, phosphate-buffered saline (PBS) solution containing 2% glutaraldehyde was gently overlaid to fix cells at room temperature for 20 min. The glutaraldehyde solution was changed to 1% Coomassie Brilliant Blue (CBB) G-250 solution (1% CBB in 50% methanol/PBS), and cells were incubated for 2 hours at room temperature. Cells were then destained with 50% methanol/PBS, and washed with PBS. Bright-field cell images were obtained using light microscopy at 20-fold magnification (BZ-X700 All-in-one Fluorescence Microscope, KEYENCE, Osaka, Japan), and color image-processing of contour segmentation was performed using Hydrid Cell Count Module internal software. The image area was divided into two regions: neurites and cell nucleus with pale and deep color segmentation, respectively. The area ratio of image extract-2 (neurites) to image extract-1 (neurites+cell nucleus) was calculated as percentage of neurite outgrowth activity.

### 2.3 *In vitro* cell viability assay

The cytotoxic effects of kCer, C16Cer, C18Cer, and C2Cer in PC12 cells were determined by conventional trypan blue exclusion assay (TBEA), lactate dehydrogenase (LDH) test, and extracellular reduction of WST-8 by electron mediator produced in cells (CCK-8). The ceramides were dissolved in 0.025%BSA/DMEM medium and added to the cultures. PC12 cells were treated with ceramides alone for 2 hours for the LDH test and 24 hours for TBEA and CCK-8, and incubated at 37 °C and 5% CO_2_.

### 2.4 CRMP2 phosphorylation assay

PC12 cell monolayers in 6-well plates were treated with NGF (100 ng/mL) for 48 hours, then replaced with fresh DMEM containing either kCer (25 μM) or Sema 3A (250 ng/mL), and incubated for 0.5–24 h. After treatment, cells were lysed in a solution of Protein Inhibitor Cocktail II (Calbiochem, Darmstadt, Germany), and protein concentrations were adjusted to 1 μg/μL according to protein determinations by a bicinchoninate protein assay kit (Nacalai Co, Kyoto, Japan). Proteins (20 μg) were separated by SDS-PAGE with 7% polyacrylamide under reducing conditions and transferred to polyvinylidene difluoride (PVDF; Bio-Rad, Hercules, CA) membranes by semi-dry blotting (Trans-Blot SD Cell, Bio-Rad, Hercules, CA). The resultant Western blots were blocked by incubation with blocking solution (BLOCKING ONE, Nacalai.Co., Kyoto, Japan) and then incubated overnight at 4 °C with primary antibodies for CRMP2, pCRMP2, and actin. The blots were incubated for 1 h with peroxidase-coupled secondary antibody (Jackson Laboratory, Bar Harbor, ME). After extensive washings, antibody binding was visualized with Chemi-Lumi One Super reagent (Nacalai.Co., Kyoto, Japan). The developed bands were quantified using JustTLC software.

### 2.5 LC–ESI–MS/MS analysis

A Prominence UFLC system (Shimadzu, Kyoto, Japan) coupled to a TripleTOF 5600 System (AB SCIEX, Foster City, CA, USA) was used for kGlcCer and kCer analyses. kGlcCer or kCer was injected onto an InertSustain column (5 μm, 2.1 i.d×150 mm, GL Science, Tokyo, Japan). Mobile phase A was acetonitrile:methanol:formic acid (95:5:0.2, v/v) containing 5 mM ammonium formate, and mobile phase B was methanol:formic acid (100:0.2, v/v) containing 5 mM ammonium formate. Samples were eluted at 0.2 mL/min through a 45 min gradient: mobile phase B, 0–5 min 0%, 5–10 min from 0% to 20%, 10–12 min hold 20%, 12–15 min from 20% to 50%, 15–22 min hold 50%, 22–27 min from 50% to 80%, 27–30 min hold, and 30–45 min from 80% to 0%. TOF–MS and MS/MS analysis were run in the positive ion mode with the following instrument parameters: curtain gas of 10, ion spray voltage of 5500 and temperature of 300 °C. The level of collision energy of each target was optimized. High resolution-multiple reaction monitoring (MRM–HR) mode was used to quantify the contents of each target. Data acquisition and analysis were performed using AnalystTF software version 1.6 (AB SCIEX).

### 2.6 Statistical analysis

The number (n) in each experimental condition is indicated in the. Data analysis was performed using the commercial program Prism 4.0 (GraphPad, San Diego, CA). When two experimental conditions were compared, statistical analysis was performed using an unpaired *t* test. Otherwise, statistical analysis was performed by one-way ANOVA followed by Tukey's Multiple Comparison post-test and Dunnet's test. A *P* value less than 0.05 was considered significant. * indicates significantly different results. Ranges of *P* values are indicated in the. NS indicates non-significant.

## 3 Results

### 3.1 Yield of EGCase I reaction

Ito M, et al. reported that globo-series glycosphingolipids are hydrolyzed to some extent by EGCase I, but that GlcCer is hardly hydrolyzed by the enzyme [10]. In spite of enzymatic hydrolysis resistance, we improved the efficiency of kGlcCer hydrolysis up to nearly 100% conversion by increasing the enzyme amount in the presence of 1% Triton X-100 (data not shown). However, because of residual toxicity of Triton X-100, we performed EGCase I reactions without any detergent, but were able to reach increased kCer production by elongation of the incubation time. However, the yield was not beyond 50% (Fig. 1B). The kCer thus prepared could be separated from unreacted kGlcCer by medium-performance liquid chromatography up to 95% of TLC-grade (Fig. 1C). One unit of EGCase I was defined as the amount of enzyme that hydrolyzes 1 μmol of kGlcCer per min as a donor substrate. In that condition, the enzyme unit per tube used was 70.3 mU (kGlcCer, 135 nmol; 250 μg of enzyme protein for 16 hour incubation). To determine the molecular species of kCer and unreacted kGlcCer after EGCase I reaction, LC–MS/MS analysis was performed using kCer and kGlcCer purified from Bligh–Dyer extraction by medium-performance liquid chromatography. We found that 6 species of ceramides were present in both kCer and kGlcCer preparations (Fig. 1D). The majority of the molecular species was a combination of sphingoid base (d18:2) and hydroxyl fatty acid (C18:0-OH) as shown in Fig. 1A, right panel. There was no remarkable difference in the molecular species between kCer and kGlcCer.

### 3.2 Cell toxicity of kCer

**Fig. 2:**
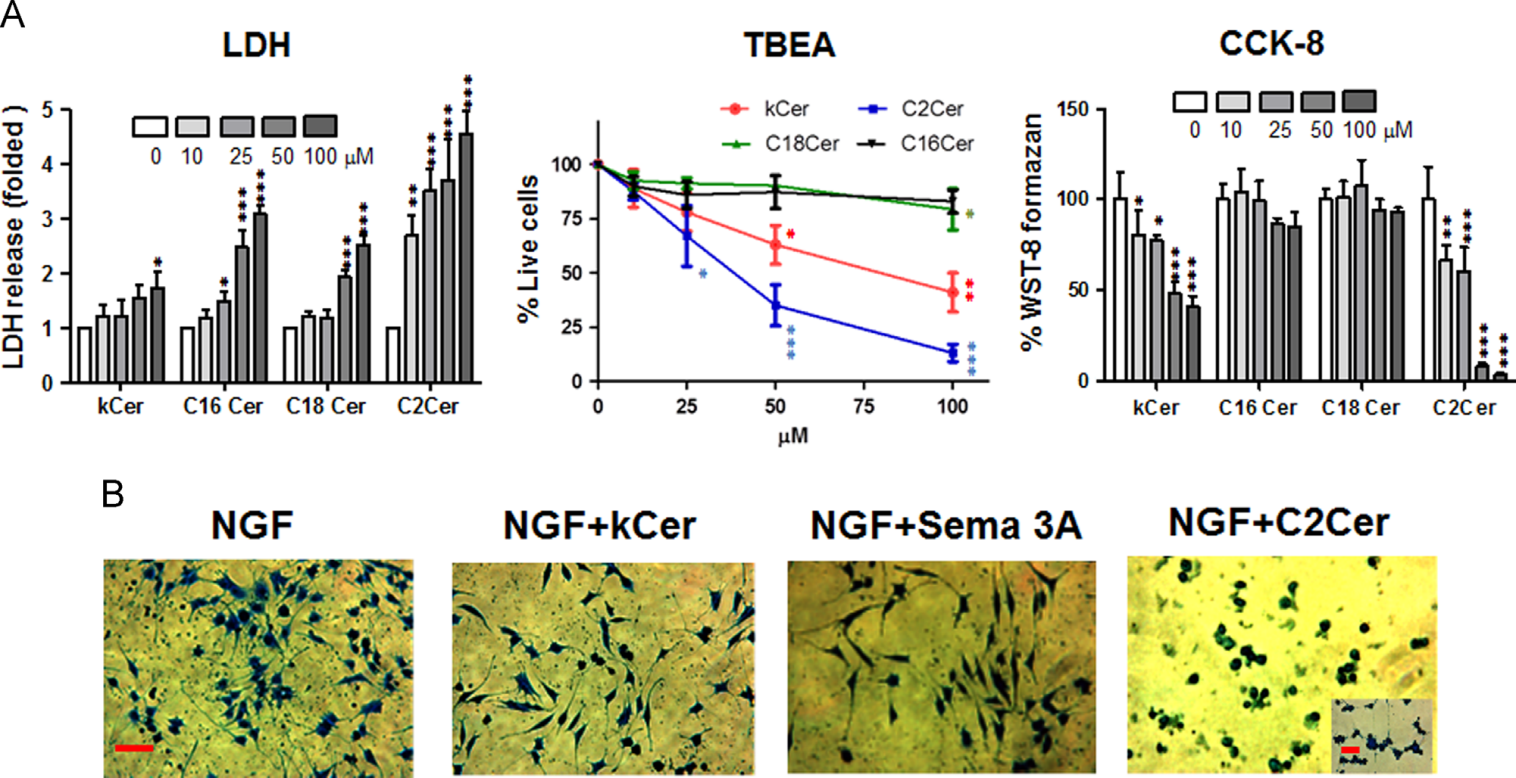
(A) Cytotoxicity of kCer, C2Cer, C16Cer, and C18Cer was determined by LDH release test (2-hour treatment), TBEA, and CCK-8 test (24-hour treatment). The data represent the means of four individual experiments with standard deviations, and analyzed by one-way ANOVA. Values are indicated by *P<0.05, **P<0.01, and ***P<0.001 vs. vehicle-treated controls (0 μM). (B) PC12 cells were cultured at 1.6×10^4^ cells/cm^2^ in a 24-well plate, and treated with kCer (50 μM), Sema 3A (250 ng/mL), or C2Cer (5 μM) in the presence of 100 ng/mL of NGF. After 2% glutaraldehyde fixation, cells were stained by 1% CBB and photographed (20× magnification). Scale bar, 100 μm.

Cytotoxicity of kCer was examined by either 2-hour treatment of PC12 cells followed by LDH release test or 24-hour treatment of PC12 cells followed by TBEA and CCK-8 tests. As shown in Fig. 2A (left panel), kCer and 3 other ceramides (C16Cer, C18Cer, and C2Cer) showed dose-dependent (from 10 μM to 100 μM) LDH release. The decreasing order of toxicity was C2Cer, C16Cer, C18Cer, and kCer. On the other hand, the percentage of live cells via TBEA was hardly influenced by C16Cer and C18Cer, but influenced weakly by kCer and strongly by C2Cer (middle panel). Similar trends of toxicity to TBEA were observed by CCK-8 test (right panel). The IC_50_ of kCer was 80 μM by TBEA, and 50 μM by CCK-8. The CCK-8 test appeared to be somewhat more sensitive than TBEA.

### 3.3 Neurite outgrowth activity and cell morphological changes

**Fig. 3:**
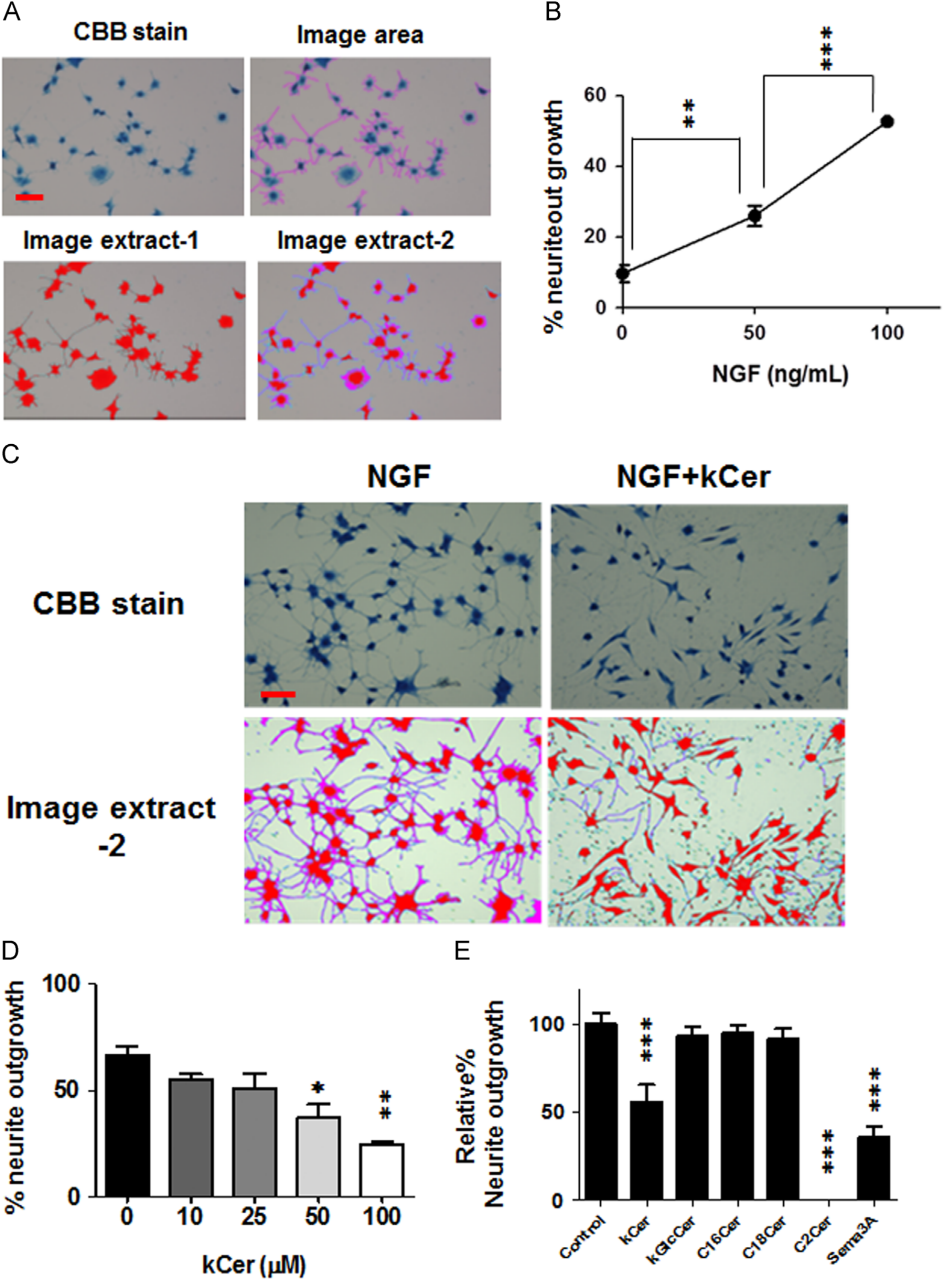
(A) Neurite outgrowth activity was measured by color image-processing. The area ratio of image extract-2 (neurites) to image extract-1 (neurites+cell nucleus) was calculated as the percentage of neurite outgrowth activity. Scale bar, 100 μm. (B) Neurite outgrowth activity correlated with NGF dose. The data represent the means of four individual experiments with standard deviations. **P<0.01, **P<0.001 vs. vehicle-treated controls (0 ng/mL NGF) respectively by one-way ANOVA. (C) Effect of 50 μM kCer on NGF-induced neurite outgrowth. Scale bar, 100 μm. (D) Inhibitory effect of kCer on NGF-induced neurite outgrowth. The data represent the means of four individual experiments with standard deviations. *P<0.05, **P<0.001 vs. vehicle-treated control (0 μM) respectively by one-way ANOVA. (E) Comparison of inhibitory effects on NGF-induced neurite outgrowth in the presence of 50 μM kCer, kGlcCer, C16Cer, C18Cer, and C2Cer. The data represent the means of four individual experiments with standard deviations. ***P<0.0001 vs. vehicle-treated control (0 μM) respectively by one-way ANOVA.

PC12 cells were induced to differentiate by NGF in the presence of kCer or C2 ceramide, and cultures were fixed and stained with CBB as described in Section 2 (Fig. 2B). NGF-induced neurite outgrowth was analyzed as color phase-based images by BXZ-700 microscopy (Fig. 3A). The image ratios of extracts 1 and 2 showed linearity with the treatment dose of NGF (Fig. 3B).

kCer treatment inhibited neurite extensions, resulting in a few remaining long neurites, and loss of short neurites (Fig. 2B, second panel, and Fig. 3C). Treatment with kCer caused a change in cell morphology, to a spindle shape, as compared with a control culture; NGF treatment alone induced a star-shaped morphology. This morphological change by co-treatment with kCer was also found with Sema 3A (Fig. 2B, third panel). On the other hand, cells treated with NGF and C2Cer showed a remarkable inhibition of neurite outgrowth (Fig. 2B, fourth panel, and Fig. 3E), resulting in cells losing long neurites with a few remaining short neurites. The cells grown in the presence of NGF and C2Cer showed round cell morphology. The neurite-inhibiting effect of kCer was dose-dependent at the range of 0 to 100 μM (Fig. 3D), and was specific to kCer at 50 μM, as compared with no inhibition by C16Cer and C18Cer (Fig. 3E). Sema 3A inhibited NGF-induced neurite outgrowth up to 35% of control (Fig. 3E), by neurite retractions with similar cell morphology as NGF+ kCer treatment (Fig. 2B).

**Fig. 4:**
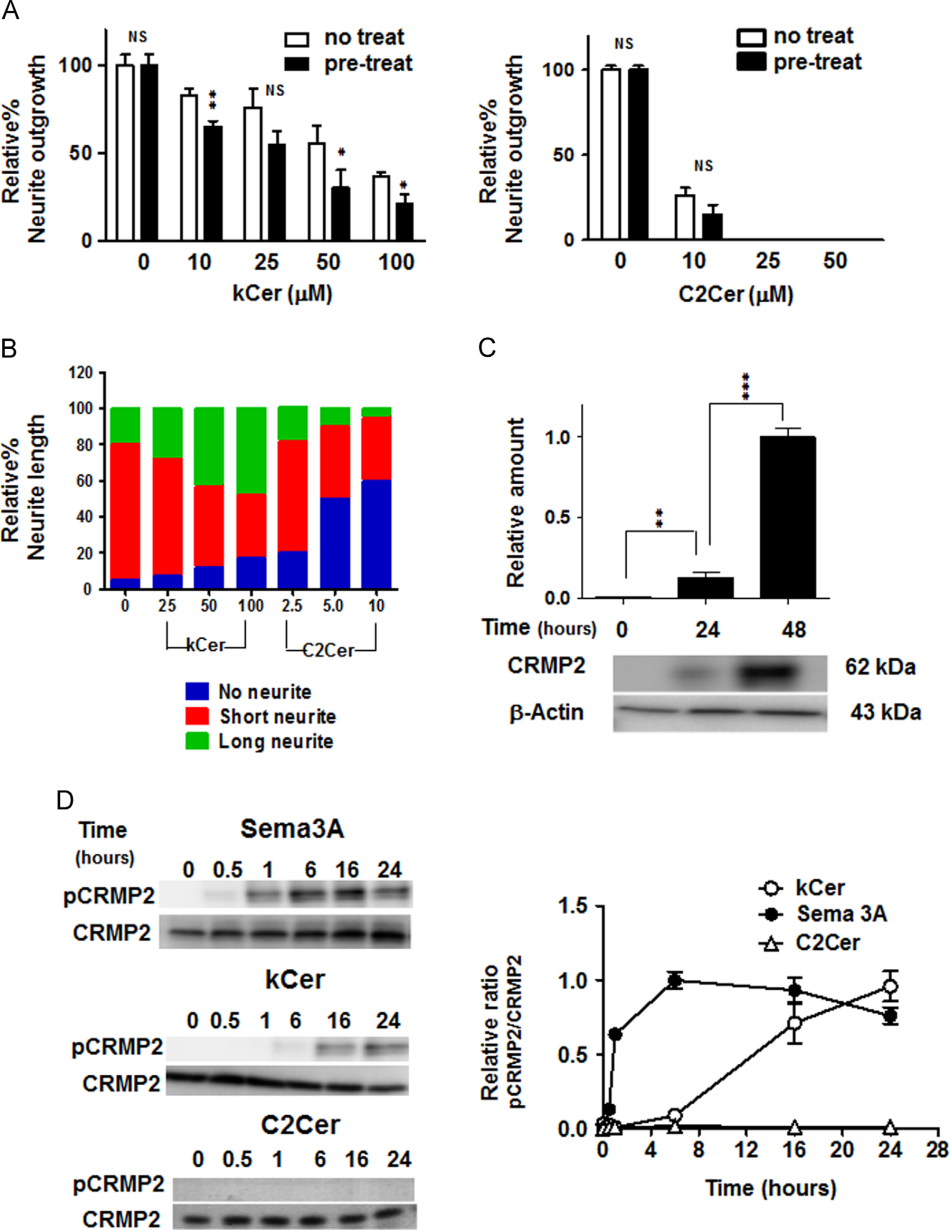
(A) Effect of pretreatment by kCer and C2Cer on NGF-induced neurite outgrowth of PC12 cells. Cells were pretreated with kCer (10–100 μM) or C2Cer (10–50 μM) with serum-free DMEM for 16 hours in a CO_2_ incubator. The media was removed and replaced with DMEM containing NGF (100 ng/mL) in the presence of kCer (10–100 μM) or C2Cer (10–50 μM), respectively. After 48 hours, cells were subjected to CBB stain and tested for neurite outgrowth activity. The data represent the means of four individual experiments with standard deviations. *P<0.05, **P<0.01 vs. no-treatment, respectively, by an unpaired *t* test. NS, not significant. (B) Effect of kCer and C2 Cer on neurite length of NGF-induced neurite outgrowth of PC12 cells. Neurite length was compared with cell body. Long neurite, more than 2-fold increase in length; short neurite, less than 2-fold increase in length; and no neurite, less than 1/10-fold original length. (C) NGF-induced CRMP2 production. PC12 cells were treated with NGF and analyzed by Western blotting using anti-CRMP2 antibody as described in Section 2. The data represent the means of three individual experiments with standard deviations. **P<0.01, **P<0.001, vs. control time (0-hour) respectively by one-way ANOVA. (D) Time course of CRMP2 phosphorylation by kCer (25 μM) and Sema 3A (250 ng/mL) treatment after NGF-induced differentiation of PC12 cells for 48 hours analyzed by Western blot (left panel). Quantitation of CRMP2 phosphorylation is calculated as the ratio of pCRMP2 to CRMP2 (right panel). The data represent the means of three individual experiments with standard deviations.

In contrast, kGlcCer showed no effect on neurite outgrowth (Fig. 3E). The effect on neurite outgrowth by pretreatment with kCer was dose-dependent, in contrast to C2Cer (Fig. 4A). The pretreatment of cells with kCer (or C2Cer) also remarkably increased the number of cells with long (or short) neurites and spindle (or star) cell shape, and was dose-dependent (Fig. 4B). The enhancing effect of kCer was also seen with treatment by Sema 3A.

### 3.4 CRMP2 phosphorylation

CRMP2 is an intracellular messenger within the Sema3A signaling cascade. Phosphorylation of CRMP2 is essential for Sema 3A activity. To examine the effects of kCer on CRMP2 phosphorylation, cells were treated with 100 ng/mL of NGF for 24 or 4 hours and analyzed by Western blotting (Fig. 4C). CRMP2 expression was induced and reached a maximal level at 48 hours. Cells were then treated with either Sema 3A (250 ng/mL) or kCer (25 μM) for 24 hours. CRMP2 phosphorylation was observed with both Sema 3A and kCer (Fig. 4D). Sema 3A induced CRMP2 phosphorylation earlier (at 0.5 hours) and reached a plateau level at 16 hours. In contrast, kCer treatment induced a more gradual rate of phosphorylation of CRMP2, although it also plateaued at 16 hours (Fig. 4D). In addition, C2Cer treatment did not show any phosphorylation of CRMP2.

## 4 Discussion

GlcCer isolated from food plants has recently been studied [15], [16] and is used for health foods and cosmetics. The sphingoid bases of GlcCer are structurally different from animal sphingosine (Fig.1A) [17]. These differences are believed to be the reason for the health-scientific effectiveness of the food plant GlcCer. The food plant sphingoid bases have also been studied, and d18:2 and t18:1 sphingoids have been demonstrated to bind peroxisome proliferator-activated receptor (PPAR)γ, up-regulating *de novo* ceramide synthesis of very long-chain fatty acids in differentiated keratinocytes [18], [19]. On the other hand, food plant ceramides that are an intermediate between GlcCer and sphingoid base have not been studied at all, and it is unclear why food plants include trace amounts of ceramides such as kCer.

In the present study, we successfully chemoenzymatically isolated a homogenous preparation of kCer, found in trace amounts in konjac extracts. This new ceramide material was shown to inhibit NGF-induced neurite outgrowth in rat adrenal pheochromocytoma cells, PC12. Because this inhibitory activity was caused by kCer but not kGlcCer, the specific hydrophilic or hydrophobic structure of the kCer molecule seemed to be essential for its activity. This activity is lacking in animal ceramides such as C16Cer and C18Cer. In addition, the mode of inhibition by kCer was characterized by suppressing short neurite sprouting and outgrowth and leaving long neurites, and was accompanied by a cell morphological change from star-shape to spindle-shape. These effects by kCer is a Sema 3A-like action that preserves the balancing of NGF/Sema 3A. There are two types of itch to be discriminated by whether the skin permeability barrier is normal or not. kCer has a Sema3A-like action, implicating that kCer may be effective for itching from abnormalities or dysfunctions of the skin permeability barrier. C2Cer strongly inhibited PC12 neurite outgrowth, and was accompanied by the disappearance of long neurites and by a round morphology (Fig. 2B, fourth panel, and Fig. 4B). Although C2Cer produced neurite retractions, CRMP2 phosphorylation was not caused by treatment with C2Cer (Fig. 4D). Thus, C2Cer action seemed to be cytotoxic rather than neurite-retractive, which is completely different from the action of kCer causing CRMP2 phosphorylation and spindle-shaped long neurite outgrowth.

In the first step of cell signaling, Sema 3A constitutes a heterotrimer complex with its receptor neuropilin1 and plexin A1 on the cell surface [20], [21]. This receptor complex activates GSK3β and Cdk5, causing CRMP2 phosphorylation downstream of Sema 3A signaling [22]. pCRMP2 no longer binds to tubulin, leading to destabilization and depolymerization of microtubules, leading to collapse of the growth cone [23]. This mechanism of growth cone stability is maintained by a quantitative relationship between NGF and Sema 3A. Our findings of cell morphological effects and CRMP2 phosphorylation by kCer suggest that it works as a Sema 3A-like agonist.

Thus, we expect that chemoenzymatically prepared konjac ceramide can be used as a beneficial alternative for Sema 3A for treating itching diseases and syndromes.

## Competing interests

The authors declare no conflict of interest.
